# Gut microbiome modulation by diet and lifestyle in kidney stone disease and autism spectrum disorder: emerging mechanisms and translational prospects

**DOI:** 10.3389/frmbi.2026.1901989

**Published:** 2026-09-22

**Authors:** Girish B. Mahajan

**Affiliations:** R & D-Microbiology Department, HiMedia Laboratories Pvt. Ltd., Mumbai, India

**Keywords:** gut microbiome, urolithiasis, autism spectrum disorder, dietary fiber, short-chain fatty acids, oxalate metabolism, polyphenols, probiotics

## Abstract

**Background:**

The gut microbiome occupies a central position in mediating the physiological consequences of diet, lifestyle, and physical activity. While this interplay is broadly recognized, its mechanistic consequences in two clinically underserved conditions—urolithiasis (kidney stone disease) and autism spectrum disorder (ASD)—remain poorly explained. Both conditions share convergent pathophysiology rooted in metabolic dysregulation, impaired epithelial barrier function, immune dysregulation, and altered microbial metabolite profiles, all of which are modifiable through dietary and behavioral interventions.

**Methods:**

This minireview synthesizes current evidence from clinical, epidemiological, and mechanistic studies to evaluate the bidirectional relationship between gut microbial ecology and disease pathogenesis in urolithiasis and ASD, with emphasis on dietary compounds, fermented foods, probiotics, and lifestyle behaviors as modulatory factors.

**Results:**

In urolithiasis, evidence implicates reduced abundance of oxalate-degrading taxa (notably *Oxalobacter formigenes*), dysbiotic short-chain fatty acid (SCFA) profiles, and urinary microbiome perturbations in stone formation and recurrence. In ASD, microbial alterations in gastrointestinal-symptomatic individuals converge on reduced butyrate-producing taxa, elevated pro-inflammatory species, and altered serotonin and gamma-aminobutyric acid (GABA) biosynthetic pathways.

**Conclusion:**

Dietary interventions—particularly high-fiber regimens, polyphenol-rich foods, fermented products, and targeted probiotic formulations—show mechanistic plausibility for microbiome restoration in both conditions. However, clinical translation is constrained by methodological heterogeneity, confounding lifestyle variables, and a paucity of longitudinal, mechanistically anchored trials. We outline a research agenda prioritizing standardized multi-omics, paired gut–urine sampling protocols, and rigorously controlled dietary interventions to bridge this gap.

## 1 Introduction

The human gut harbors approximately 3.8 × 10¹³ microbial cells, encoding a metabolic repertoire that dwarfs the host genome by two orders of magnitude (Sender et al., 2016; Harmsen and de Goffau, 2016). This microbial community—shaped decisively by dietary composition, physical activity, sleep architecture, and psychosocial factors—functions as a dynamic interface between environmental exposures and systemic physiology. The past decade has witnessed remarkable progress in mapping how specific dietary substrates (notably fermentable fibers, polyphenols, and fermented food matrices) and lifestyle determinants selectively enrich or deplete microbial taxa with functional consequences spanning metabolic, immunological, and neurological domains (Sonnenburg and Bäckhed, 2016; Ding et al., 2019).

Against this background, two clinically important conditions stand out for the relative scarcity of microbiome-driven mechanistic understanding despite carrying significant global burden. Urolithiasis affects approximately 12% of the global population over a lifetime (Pearle et al., 2014), with recurrence rates exceeding 50% within five years (Pearle et al., 2014) and with risk influenced by dietary determinants, including oxalate, calcium, and purine intake (Pearle et al., 2014). Autism spectrum disorder (ASD) is estimated to affect 1 in 36 children in the United States and is characterized by high rates of gastrointestinal (GI) comorbidity (Maenner et al., 2023)—present in up to 70% of affected individuals (De Angelis et al., 2015)—that amplify behavioral symptoms and reduce quality of life. Both conditions sit at the intersection of metabolic regulation, epithelial barrier integrity, immune activation, and host–microbe signaling, rendering them compelling targets for microbiome-directed dietary and lifestyle intervention (Round and Mazmanian, 2009).

This minireview examines the current state of evidence on gut microbiome modulation in urolithiasis and ASD through the lens of dietary compounds, lifestyle behaviors, and physical activity, consistent with the scope of the present Research Topic. We specifically address mechanistic pathways linking microbial function to clinical endpoints, critically evaluate intervention evidence, and propose a framework for advancing translational research in both domains.

Importantly, this review does not suggest that urolithiasis and autism spectrum disorder (ASD) share the same disease mechanisms. Rather, both conditions may involve a common upstream feature: depletion of similar butyrate-producing gut bacteria, particularly *Faecalibacterium prausnitzii*, *Roseburia* spp., and *Blautia* spp. However, the metabolic effects of this dysbiosis differ depending on the affected organ system.

A shared dietary approach—using fermentable fiber to increase short-chain fatty acid production—may therefore be relevant to both conditions through the same microbial pathway. Disease-specific mechanisms remain clearly distinct: oxalate degradation and urinary microbiome changes in urolithiasis, and serotonergic, GABAergic, and neuroimmune pathways in ASD. These are discussed separately and are not presented as equivalent mechanisms. The shared upstream pathways and distinct disease-specific mechanisms are summarized schematically in **Figure 1**.

**Figure 1:**
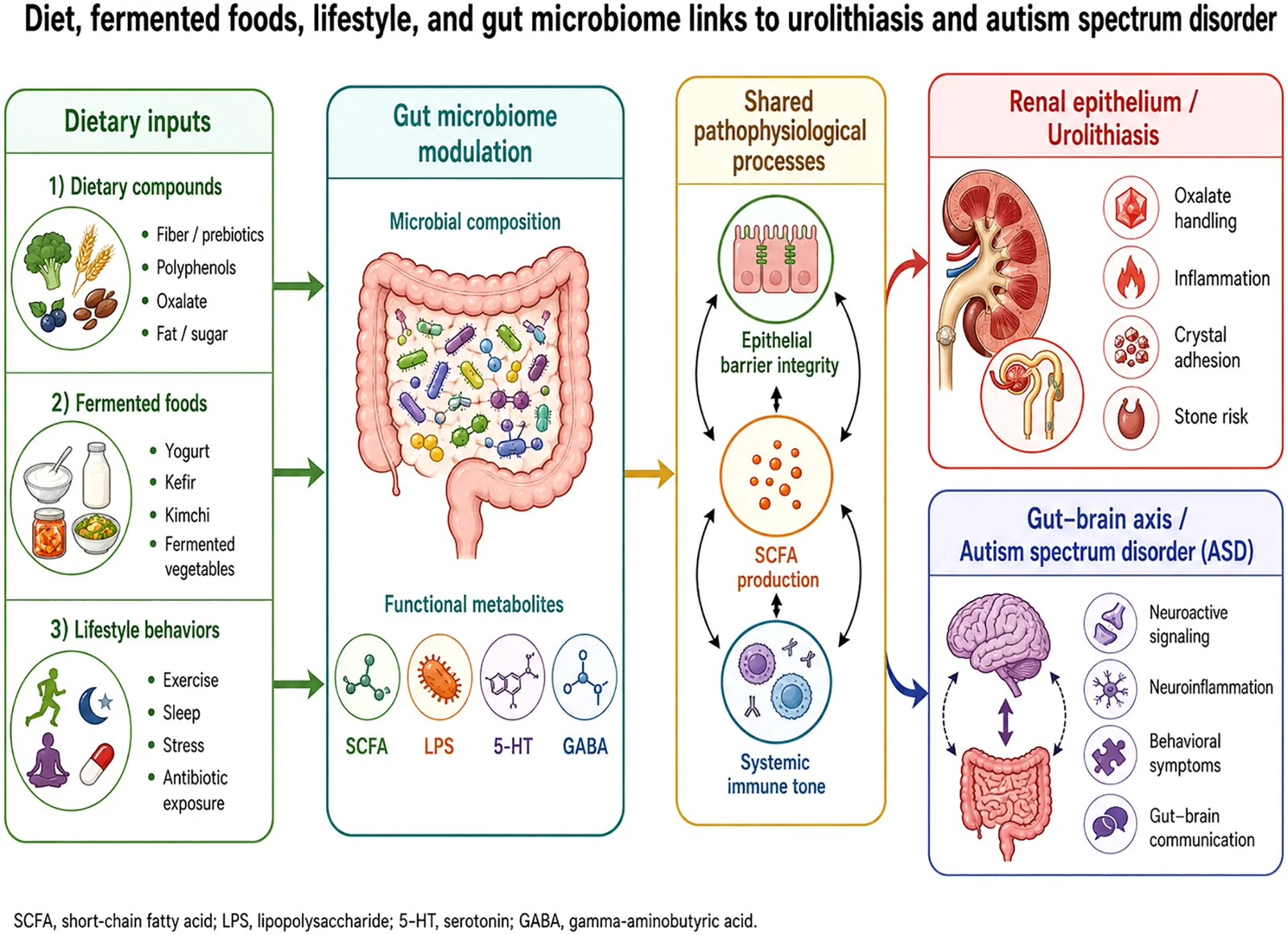
Conceptual framework linking dietary compounds, fermented foods, and lifestyle behaviors to gut microbiome modulation and disease-relevant outcomes in urolithiasis and autism spectrum disorder. Dietary inputs (left) shape gut microbial composition and functional metabolite output (center), which converge on shared pathophysiological processes—epithelial barrier integrity, SCFA production, and systemic immune tone—with divergent but overlapping organ-level consequences in renal epithelium (urolithiasis, upper right) and the gut–brain axis (ASD, lower right). SCFA, short-chain fatty acid; LPS, lipopolysaccharide; 5-HT, serotonin; GABA, gamma-aminobutyric acid.

## 2 The gut microbiome in urolithiasis: beyond a single protective species

### 2.1 Epidemiological and compositional evidence

The association between gut microbiome composition and urolithiasis has historically been anchored to *Oxalobacter formigenes* (Kaufman et al., 2008), a strict anaerobe that colonizes the human colon and enzymatically degrades intestinal oxalate via the enzymes oxalyl-CoA decarboxylase and formyl-CoA transferase (Miller et al., 2022). Epidemiological studies consistently link *O. formigenes* colonization to reduced urinary oxalate excretion and lower stone recurrence risk, with colonization-negative individuals exhibiting significantly higher 24-hour urinary oxalate levels (Kaufman et al., 2008). However, a simplistic ‘protective species’ model fails to capture the full microbiome–oxalate nexus. Cross-sectional 16S rRNA sequencing studies demonstrate that stone-formers harbor altered microbial communities characterized by reduced overall diversity, decreased *Bacteroidetes* and increased *Firmicutes* abundance, and depletion of SCFA-producing genera including *Faecalibacterium*, *Roseburia*, and *Butyrivibrio*, independent of *O. formigenes* status (Mariat et al., 2009).

### 2.2 Mechanistic pathways: oxalate handling, SCFAs, and epithelial integrity

Intestinal oxalate homeostasis is governed by several intersecting mechanisms beyond direct degradation. Epithelial transport of oxalate via the SLC26 family of anion exchangers (particularly SLC26A3 and SLC26A6) mediates bidirectional flux between the gut lumen and portal circulation (Miller et al., 2022); microbial SCFAs modulate expression of these transporters, thereby influencing intestinal oxalate secretion and urinary oxalate load. Butyrate concurrently reinforces intestinal tight junction integrity through histone deacetylase (HDAC) inhibition and NF-κB pathway suppression, limiting paracellular oxalate permeation that is exacerbated in dysbiotic states (Konig et al., 2016).

Several butyrate-producing taxa are relevant to oxalate homeostasis. *Faecalibacterium prausnitzii*, one of the major butyrate producers in the healthy human colon, is consistently depleted in calcium oxalate stone formers (Ticinesi et al., 2018). It produces butyrate mainly through the butyryl-CoA CoA-transferase pathway.

In a controlled animal model, short-chain fatty acids reduced urinary oxalate and renal calcium oxalate deposition by regulating intestinal oxalate transporters. However, the effects differed among individual SCFAs: butyrate reduced expression of the oxalate-absorbing transporter SLC26A3, whereas acetate and propionate increased expression of the oxalate-secreting transporter SLC26A6 (Liu et al., 2021).

*Roseburia intestinalis* and *Roseburia hominis*, members of the Lachnospiraceae family, also produce butyrate through the butyryl-CoA CoA-transferase pathway. Reduced abundance of *Roseburia* sp. has been reported in some stone-forming populations. Fermentable fibers such as arabinoxylan and inulin may support *Roseburia* and other butyrate-producing bacteria, although responses vary by fiber type, microbial species, and individual microbiome. This provides a plausible microbial link to the lower kidney-stone risk associated with adherence to the Dietary Approaches to Stop Hypertension (DASH)-style diet (Taylor et al., 2009), although that study did not directly evaluate microbiome changes.

*Anaerobutyricum hallii* and *Anaerostipes hadrus* further support colonic butyrate production by converting lactate and acetate generated by other fermenting bacteria into butyrate. This cross-feeding provides functional redundancy within the butyrate-producing ecosystem.

*Butyrivibrio fibrisolvens* is also a butyrate-producing bacterium; however, its proposed ability to degrade oxalate has not been sufficiently confirmed, particularly in humans, and should not presently be considered an established mechanism of stone prevention.

Collectively, depletion of these interconnected butyrate-producing bacteria may impair oxalate homeostasis through reduced SCFA production, altered intestinal oxalate transport, and loss of microbial metabolic cooperation.

The urinary microbiome presents an additional, largely underexplored dimension (Miller et al., 2022). Emerging evidence from mid-stream clean-catch and catheter-collected specimens analyzed by expanded quantitative urine culture (EQUC) and metagenomics reveals distinct microbial communities in stone-formers versus non-formers, dominated by Gram-negative uropathogens and characterized by urease-producing species capable of generating struvite nuclei (Miller et al., 2022). Integrating gut and urinary microbiome data in the same cohort—a methodological gap in virtually all published studies—represents a high-priority research need (Miller et al., 2022; Bokulich et al., 2018; Pearle et al., 2014).

### 2.3 Dietary and lifestyle modulators

High dietary fiber intake predictably shifts gut microbial composition toward greater SCFA output and enriches oxalate-handling taxa (Sonnenburg and Bäckhed, 2016; Wu et al., 2011). The DASH diet—characterized by high fruit, vegetable, and whole grain content with restricted sodium and animal protein—reduces urinary calcium-oxalate supersaturation and has been associated with reduced stone incidence in large prospective cohorts (Taylor et al., 2009), plausibly through microbiome-mediated as well as direct dietary mechanisms (Sonnenburg and Bäckhed, 2016). Plant-derived polyphenols modulate the microbiome with notable selectivity: ellagitannins from pomegranate, berries, and walnuts are converted by colonic microbiota to urolithins A and B, which exhibit anti-inflammatory and epithelial-protective properties in preclinical models relevant to nephro-urological epithelium (Espín et al., 2013).

Fermented foods—yogurt, kefir, fermented vegetables—contribute live oxalate-metabolizing lactic acid bacteria (LAB) strains, including select Lactobacillaceae that possess functional oxalate-degrading enzyme systems. While the colonization efficiency of exogenous LAB strains in adult gut ecosystems is modest and transient, regular consumption may provide clinically meaningful oxalate flux reduction, particularly in antibiotic-exposed populations where indigenous oxalate-degraders are suppressed (Vangay et al., 2015). Probiotic intervention studies using *Lactobacillus acidophilus* and *Bifidobacterium animalis* formulations have demonstrated modest but statistically significant reductions in 24-hour urinary oxalate in randomized controlled trials, though effect sizes and durability remain inconsistent across studies.

Physical activity independently shapes microbial community structure, enriching taxa associated with greater microbial metabolic flexibility. Sedentary behavior, conversely, associates with reduced microbial diversity—a pattern overlapping substantially with the dysbiotic signature observed in stone-forming populations. Obesity and metabolic syndrome, both exacerbated by sedentary behavior, potentiate uric acid stone formation through increased urinary uric acid excretion and reduced urinary pH; the microbiome mediates a portion of this risk through altered purine catabolism by gut bacteria (Taylor et al., 2005).

### 2.4 Stone-type-specific microbiome relevance

The mechanistic relationship between gut dysbiosis and urolithiasis is not phenotype-agnostic, and generalizing findings across all stone subtypes risks obscuring clinically meaningful distinctions. Calcium oxalate stones, the dominant phenotype (~75–80% of cases), have the richest gut microbiome evidence base: depletion of *O. formigenes*, SCFA-producing *Faecalibacterium*, *Roseburia*, and *Butyrivibrio* genera, impaired SLC26A6-mediated oxalate secretion, and butyrate-deficient tight-junction compromise are all specifically documented in this subtype (Ticinesi et al., 2018; Miller et al., 2022). Uric acid stones (~10–15%) operate through a distinct microbiome axis: gut bacterial purine catabolism reduces luminal urate available for absorption, and depletion of *Akkermansia muciniphila* and Christensenellaceae — taxa inversely correlated with BMI and insulin resistance — potentiates the metabolic syndrome phenotype that drives low urinary pH and hyperuricosuria (Taylor et al., 2005; Kim et al., 2022). Struvite stones (~5–15%) are infection-driven, with urease-producing uropathogens (*Proteus mirabilis*, *Klebsiella pneumoniae*) as the direct cause; the gut microbiome is implicated indirectly through barrier dysfunction-facilitated uropathogen translocation and impaired colonization resistance, a mechanistic link that remains speculative and under-studied. Cystine urolithiasis (<3%), being monogenic in etiology (SLC3A1/SLC7A9 mutations), has no established gut microbiome involvement, though gut bacterial methionine catabolism represents an unexplored theoretical pathway. Future microbiome studies in urolithiasis must stratify explicitly by stone type — a methodological imperative that is currently unmet in the vast majority of published cohort studies (Pearle et al., 2014).

## 3 The gut microbiome in autism spectrum disorder: signal within the noise

### 3.1 Gastrointestinal comorbidity and microbial associations

The intersection of ASD and gut dysfunction is clinically conspicuous and mechanistically fertile (De Angelis et al., 2015). GI symptoms—constipation, diarrhea, abdominal pain, and bloating—affect a substantial proportion of ASD-diagnosed individuals and correlate with greater behavioral symptom severity, reduced adaptive functioning, and impaired sleep (De Angelis et al., 2015). The gut–brain axis, encompassing vagal afferents, enteric nervous system signaling, immune-mediated cytokine trafficking, and metabolite-driven neuro-endocrine pathways, constitutes the likely bidirectional conduit through which gut dysbiosis amplifies neurobehavioral phenotypes (Petra et al., 2015).

Metagenomic and 16S rRNA profiling studies consistently identify altered microbial communities in ASD cohorts, though the specific taxa implicated vary considerably across studies due to geographic differences, dietary habits, antibiotic exposure histories, and methodological heterogeneity in sequencing platforms and analytical pipelines. Broadly reproducible findings include: reduced abundance of butyrate-producing taxa (*Faecalibacterium prausnitzii*, *Roseburia intestinalis*, and *Blautia* species) (Sharon et al., 2019); enrichment of *Clostridia* cluster members producing p-cresol—a uremic toxin associated with behavioral deterioration in ASD (Finegold et al., 2010); reduced abundance of *Bifidobacterium* spp. (De Angelis et al., 2015) and *Akkermansia* spp. (Sharon et al., 2019); and elevated *Desulfovibrio* species (Finegold et al., 2010), which are sulfate-reducing bacteria capable of generating hydrogen sulfide with pro-inflammatory properties.

### 3.2 Mechanistic pathways: metabolites, barrier function, and neuroimmune signaling

The mechanistic plausibility of microbiome involvement in ASD neurobiology rests on several converging metabolic pathways. Butyrate, produced by colonic fermentation of dietary fiber by anaerobic bacteria, crosses the blood–brain barrier via monocarboxylate transporters and exerts direct neurotrophic effects, modulates microglial activation states, and suppresses neuroinflammation through HDAC inhibition (Stilling et al., 2016). Butyrate deficiency in ASD-associated dysbiosis may therefore contribute to the low-grade neuroinflammatory milieu and altered synaptic pruning patterns documented in post-mortem and neuroimaging studies (Sharon et al., 2019).

Tryptophan catabolism represents another microbiome–brain interface of particular relevance. Approximately 90–95% of systemic serotonin (5-hydroxytryptamine, 5-HT) is synthesized in enterochromaffin cells of the gut mucosa under microbial regulatory influence (Petra et al., 2015); gut microbiota modulates host tryptophan hydroxylase 1 expression and L-tryptophan bioavailability, shaping both peripheral serotonin tone and central 5-HT precursor availability (Petra et al., 2015). Microbial perturbations in ASD may therefore impair serotonergic signaling with downstream consequences for social behavior, sensory processing, and repetitive behavior domains. Analogously, microbial synthesis of GABA—primarily by *Lactobacillus* and *Bifidobacterium* species utilizing glutamate decarboxylase—is reduced in ASD-associated microbial profiles, potentially contributing to excitatory–inhibitory imbalance (Petra et al., 2015).

Impaired intestinal barrier function—documented by elevated fecal zonulin, reduced claudin-1 and occludin expression, and heightened lactulose/mannitol ratios in a subset of ASD individuals—facilitates translocation of microbial lipopolysaccharides (LPS), flagellin, and other pattern recognition receptor ligands into systemic circulation. Systemic LPS burden activates Toll-like receptor 4 (TLR4) on microglia, amplifying neuroinflammatory signaling (Round and Mazmanian, 2009). Diet-induced microbiome shifts can measurably alter intestinal permeability within weeks, suggesting that nutritional interventions represent a realistic strategy for barrier restoration in ASD (Konig et al., 2016).

### 3.3 Dietary interventions and probiotic strategies

The gluten-free, casein-free (GFCF) diet has attracted substantial parental and clinical interest in ASD, based on the opioid peptide hypothesis positing that incomplete digestion of gluten and casein generates bioactive exorphins with neuromodulator effects (Millward et al., 2008). Controlled evidence for behavioral benefit remains limited and methodologically contested; microbiome studies suggest that GFCF diets reduce overall dietary fiber diversity—a concern in a population already prone to dietary selectivity—which may paradoxically worsen dysbiosis rather than ameliorate it (Millward et al., 2008). More nutritionally complete approaches centered on increasing fermentable fiber, Mediterranean-pattern food variety, and polyphenol-rich plant foods offer a more evidence-aligned dietary framework for ASD microbiome modulation (Sonnenburg and Bäckhed, 2016).

Probiotic intervention trials in ASD have employed a range of formulations, predominantly *Lactobacillus acidophilus*, *L. rhamnosus* GG, and multi-strain combinations including *Bifidobacterium longum* and *Streptococcus thermophilus* (Thomas et al., 2014). Meta-analyses through 2024 report modest, heterogeneous improvements in GI symptom severity, some behavioral parameters (irritability, social communication scores), and reduction in urinary p-cresol—though effect sizes are generally small, study durations range from 6–16 weeks (likely insufficient for durable colonization shifts), and behavioral outcomes are burdened by subjectivity and placebo response rates of 20–35% (Thomas et al., 2014). Synbiotic formulations co-administering prebiotic fructo-oligosaccharides or inulin alongside probiotic strains demonstrate more consistent microbiome modulation in pilot studies, representing a more mechanistically coherent intervention strategy.

Emerging interest surrounds fecal microbiota transplantation (FMT) in ASD, catalyzed by an open-label study by Kang et al. (2019), demonstrating durable microbiome shifts and behavioral improvements at two-year follow-up after a standardized FMT protocol. A subsequent double-blind, randomized controlled trial corroborated modest GI and behavioral benefits. While not yet ready for clinical adoption, FMT provides a methodological opportunity for the field: rigorously controlled FMT studies with comprehensive multi-omic profiling can establish causal relationships between microbiome composition and ASD-relevant outcomes that no dietary intervention study can easily achieve (Sharon et al., 2019).

## 4 Convergent pathophysiological themes and shared dietary levers

Examining urolithiasis and ASD together through the lens of shared microbial drivers and dietary levers — rather than common disease mechanisms or clinical presentations — reveals striking convergences in putative microbiome-mediated upstream pathways (**Table 1**). Both conditions are associated with reduced microbial diversity and depleted butyrate-producing communities. Both feature impaired epithelial barrier integrity—at the intestinal level—that amplifies systemic inflammatory burden (Round and Mazmanian, 2009). Both are exacerbated by dietary patterns low in fermentable fiber and high in ultra-processed foods, which suppress microbial metabolic flexibility and reduce the production of protective metabolites including SCFAs, urolithin A, and equol.

**Table 1:** Comparative overview of gut microbiome alterations, mechanistic pathways, and dietary/lifestyle modulators in urolithiasis and autism spectrum disorder.

Parameter	Urolithiasis	Autism spectrum disorder
Key microbial alterations	Reduced *O. formigenes, Faecalibacterium, Roseburia*; ↑ Firmicutes/Bacteroidetes ratio; altered urinary microbiome	Reduced *F. prausnitzii, Bifidobacterium, Akkermansia*; ↑ *Desulfovibrio*, *Clostridium* cluster members; elevated p-cresol producers
Central metabolic pathway	Oxalate degradation, SCFA-mediated anion transporter regulation, epithelial barrier maintenance	SCFA-mediated neuroinflammation suppression, tryptophan–serotonin axis, GABAergic signaling, LPS translocation
Shared pathophysiology	Reduced microbial diversity; butyrate deficiency; impaired intestinal epithelial integrity; systemic low-grade inflammation	Reduced microbial diversity; butyrate deficiency; impaired intestinal epithelial integrity; systemic low-grade inflammation
Dietary modulators	DASH diet; high fiber; reduced oxalate/animal protein; fermented foods (oxalate-metabolizing LAB); polyphenols (ellagitannins → urolithins)	Mediterranean-type diet; high fermentable fiber; polyphenols (quercetin, resveratrol, curcumin); fermented foods; avoidance of ultra-processed foods
Probiotic/synbiotic evidence	Modest ↓ in urinary oxalate with select Lactobacillaceae; synbiotics show more durable shifts; *O. formigenes* supplementation trials ongoing	Multi-strain probiotics → modest GI and behavioral benefit; synbiotics show greater microbiome modulation; FMT early-stage but causally informative
Lifestyle factors	Physical activity ↑ microbial diversity; sedentary behavior + obesity → uric acid stone risk; antibiotic disruption of oxalate-degrading colonization	Physical activity → butyrate-producer enrichment; sleep disruption worsens dysbiosis; medication confounders (PPI, antibiotics)
Key evidence gaps	Paired gut–urinary microbiome data; stratification by stone type; longitudinal designs post-intervention; antibiotic covariate capture	Causal vs. epiphenomenal signals; objective behavioral endpoints; dietary selectivity confounder control; long-term intervention durability

Polyphenols warrant particular attention as transdiagnostic dietary modulators. Quercetin, resveratrol, and curcumin—abundant in plant-rich diets—are extensively bio-transformed by gut microbiota into bioactive metabolites with anti-inflammatory, neuroprotective, and potentially crystallization-inhibiting properties (Espín et al., 2013; Sonnenburg and Bäckhed, 2016). The biotransformation efficiency, and thus clinical benefit, is itself microbiome-dependent, creating an interplay in which dietary polyphenol intake and microbial diversity are mutually reinforcing. High physical activity similarly enriches microbial diversity in both healthy populations and chronic disease cohorts, with mechanistic data implicating lactate-based cross-feeding that favors butyrate producers—an effect directly relevant to both conditions reviewed here.

## 5 Critical appraisal and future research priorities

Despite mechanistic plausibility, translational progress in both domains is constrained by systematic methodological limitations. Study heterogeneity in 16S variable region selection, sequencing depth, bioinformatics pipeline, and reference database versions renders cross-study comparison unreliable. Most dietary intervention studies fail to account for baseline microbiome composition as a treatment response modifier—a critical omission given that individuals with greater baseline dysbiosis likely respond differently to the same intervention. Sample sizes are frequently underpowered for multi-omic endpoint analysis, and longitudinal designs with follow-up exceeding six months are rare (Bokulich et al., 2018).

For urolithiasis specifically, the near-complete absence of paired gut–urinary microbiome sampling in the same subjects limits understanding of the gut-to-kidney microbial relay. Stone type (calcium oxalate, uric acid, struvite, cystine) profoundly alters the relevant microbiome-metabolite landscape and must be explicitly incorporated in study stratification (Pearle et al., 2014). Antibiotic exposure—a potent disruptor of *O. formigenes* colonization—is infrequently captured as a covariate despite its well-documented impact on oxalate handling (Vangay et al., 2015).

For ASD, the principal challenge is separating microbiome signatures that are causal from those that are epiphenomenal to dietary selectivity, sedentary behavior, and medication use (especially proton pump inhibitors and antibiotics). Matched co-twin and sibling designs, and stratified analyses by GI symptom presence/absence, offer methodological strategies to improve causal inference. Endpoint selection in behavioral trials must move beyond global impression scales toward objective, neurophysiologically anchored measures—event-related potentials, eye-tracking metrics, and actigraphy-derived behavior variables—that are less susceptible to placebo inflation.

A high-priority research agenda for both domains should incorporate: (1) prospective longitudinal cohort studies with quarterly biospecimen collection including stool, urine, and plasma for integrated metabolomics (Bokulich et al., 2018); (2) standardized dietary assessment instruments sensitive to fiber subtypes and polyphenol class intake (Wu et al., 2011); (3) randomized dietary intervention trials powered for microbiome primary endpoints with clinical outcomes as secondary endpoints (Thomas et al., 2014); (4) mechanistic studies employing germ-free animal colonization and human organoid models to establish causality for specific taxa-metabolite-phenotype relationships (Sharon et al., 2019); and (5) open data standards enabling federated meta-analysis across geographic populations (Bokulich et al., 2018).

## 6 Conclusion

Urolithiasis and autism spectrum disorder represent two clinically significant, microbiome-tractable conditions where targeted dietary and lifestyle intervention holds genuine promise—yet where the evidence base remains in a productive but insufficiently mature state. The mechanistic framework linking dietary compounds, fermented foods, physical activity, and gut microbial function to oxalate dysmetabolism in kidney stone disease and to gut–brain neuroimmune dysregulation in ASD is scientifically coherent and increasingly supported by experimental data. What the field now urgently requires is methodological rigor: standardized protocols, longitudinal designs, functional multi-omics, and mechanistically anchored clinical endpoints. Advancing this agenda will transform a landscape of promising associations into decision-grade evidence capable of informing individualized nutritional and behavioral strategies for microbiome optimization in both conditions.
